# Age-Related Changes in the Ruminal Microbiota and Their Relationship With Rumen Fermentation in Lambs

**DOI:** 10.3389/fmicb.2021.679135

**Published:** 2021-09-20

**Authors:** Xuejiao Yin, Shoukun Ji, Chunhui Duan, Peizhi Tian, Sisi Ju, Hui Yan, Yingjie Zhang, Yueqin Liu

**Affiliations:** College of Animal Science and Technology, Hebei Agricultural University, Baoding, China

**Keywords:** sheep, rumen microbiota, volatile fatty acid, microbiota crude protein, blood metabolites

## Abstract

The rumen microbiota is vital for the health and growth performance of the host animal, mainly due to its role in the fermentation of ingested feed within the rumen. Attaining a better understanding of the development of the bacterial community and fermentation in the rumen can provide the theoretical basis for regulating feed utilization. This study analyzed the development of rumen bacteria in lambs from birth to 4 months of age using 16S-rRNA amplicon sequencing data and studied its relationship with ruminal fermentation. Serum levels of metabolites were monitored at 30, 60, 90, and 120 days of age, and the RandomForest approach was used to determine age-related changes in rumen bacteria. Levels of blood metabolites, ruminal fermentation, the rumen bacterial community and its functions were all affected by the age of the lambs (*P* < 0.05). Based on the Bray-Curtis distance within the age groups of the rumen microbiota, the similarity increased sharply after the lambs were weaned at 60 days of age (*P* < 0.05). The similarity between the samples collected from birth to 90 days of age and those collected at 120 days of age, increased after 20 days of age, reaching a maximum at 90 days vs. 120 days (*P* < 0.05). Some age-associated changes in the microbial genera were correlated with changes in the concentrations of volatile fatty acids and the levels of microbial crude protein in the rumen, including positive correlations between main volatile fatty acids and the genera of *Prevotella 1*, *Lachnospiraceae NK3A20 group*, *Ruminococcus gauvreauii group*, *Ruminococcaceae UCG-014*, and *Ruminococcus 2* (*P* < 0.05). These results indicated that the microbial community and the function of rumen was not well-established before 20 days of age, so there is a degree of plasticity in the rumen bacterial community during the first 20 days of post-natal development in lambs, and this might provide an opportunity for interventions to improve rumen fermentation and, thus, increase their growth performance.

## Introduction

The rumen is vital for the metabolism, immunity, and health of the ruminant, which hosts a complex and dynamic ecosystem containing a great diversity of microbiota. The ruminant is completely dependent on the microbial community to ferment the ingested material (Huws et al., 2018; Morais and Mizrahi, 2019). The fermentation process provides most of the energy and protein to meet the needs of the host, mainly in the form of short-chain fatty acids (SCFAs) and microbial proteins (Weimer, 2015). Therefore, to increase feed utilization efficiency and improve animal health and production, we need to thoroughly understand the development of the nutrient organ-rumen.

The colonization of the gastrointestinal tract of newborns starts at birth, and the succession process continues until the microbiota reaches a stable state later in life. Young animals such as lambs can be considered to be non-ruminants from a nutritional standpoint (Drackley, 2008). Various factors, such as diet and the surrounding environment, may influence microbial colonization (Zhu et al., 2018). Many events or disturbances may affect the rumen microbiota composition. These modifications may be more significant in successive colonization processes when the microbiota is less stable and relatively simple (Saro et al., 2018). Compared to less mature communities, a diverse and well-established microbial community within the rumen has higher resilience and is more resistant to disturbances. The composition and function of the microbiota will tend to return to their pre-treatment status once the disturbance ceases (Shade et al., 2012; Kittelmann et al., 2014). However, in young ruminants, the influence of alterations may persist for some time after the end of the modification. Saro et al. (2018) showed that a long-term early life intervention can affect the composition of the rumen microbial community and persist weeks after the intervention. Abecia et al. (2018) showed that the treatment effect persisted after the end of the modification in the early life of kid goats. Currently, many ways to change the rumen fermentation through early life microbiota have been studied, including feed styles (Zhang et al., 2019), weaning method (Mao et al., 2021), rumen fluid inoculation (Yu et al., 2020), and different additives (Lin et al., 2019; Wang et al., 2021). Thus, the rudimentary status of the ruminants’ microbiota in the early period provides an opportunity for human intervention in the rumen colonization process. However, further study is essential to gain a better understanding of the rumen colonization process and the crucial period when it can be easily moderated. There is a dearth of study on the development of the rumen as a nutrient organ and the changing process of ruminal fermentation.

In order to determine an appropriate developmental stage for the timing of the interventions, we hypothesized that the rumen bacterial communities and ruminal fermentation would change significantly over the growing period of lambs and therein persisted the key microbiota required in this process. The present study was conducted to examine the changes of ruminal fermentation and the process of microbial colonization in the rumen. This information will improve our understanding of the microbial ecology in the rumen and will strengthen the methodology for modulating the microbial community to improve the productivity and health of ruminants.

## Materials and Methods

This study was conducted under the guidance of the Animal Care and Use Committee of Hebei Agricultural University (approval number: YJ201825).

### Animals, Diets, and Experimental Design

The study was conducted between January and August 2019 at an experimental sheep farm facility in Hengshui, China. All sheep used in the study were of the Hu sheep breed. Twenty pregnant ewes (44.7 ± 1.7 kg BW, mean ± SD) carrying a singleton lamb were identified by ultrasonic examination (TTY2018, Liaochen, China). They were kept in individual pens (3.0 × 0.8 m) with free access to feed and water.

Ten newborn female lambs were included in this study (2.87 ± 0.28 kg BW). These lambs were raised according to conventional housing and growth practices used in our experimental facility. Briefly after birth, the lambs remained with their dams in individual pens and had free access to a starter commercial compound from day 15. On day 60, the lambs were weaned and offered a mixed ration at 0730 and 1,500 h each day with approximately 5% feed refusal. The ingredients and nutrient composition of the diets are provided in Supplementary Table 1. Fresh water was freely available throughout the experimental period. Unhealthy individuals (*n* = 3) were removed at 120 days of age.

### Sample Collection

**FIGURE 1:**
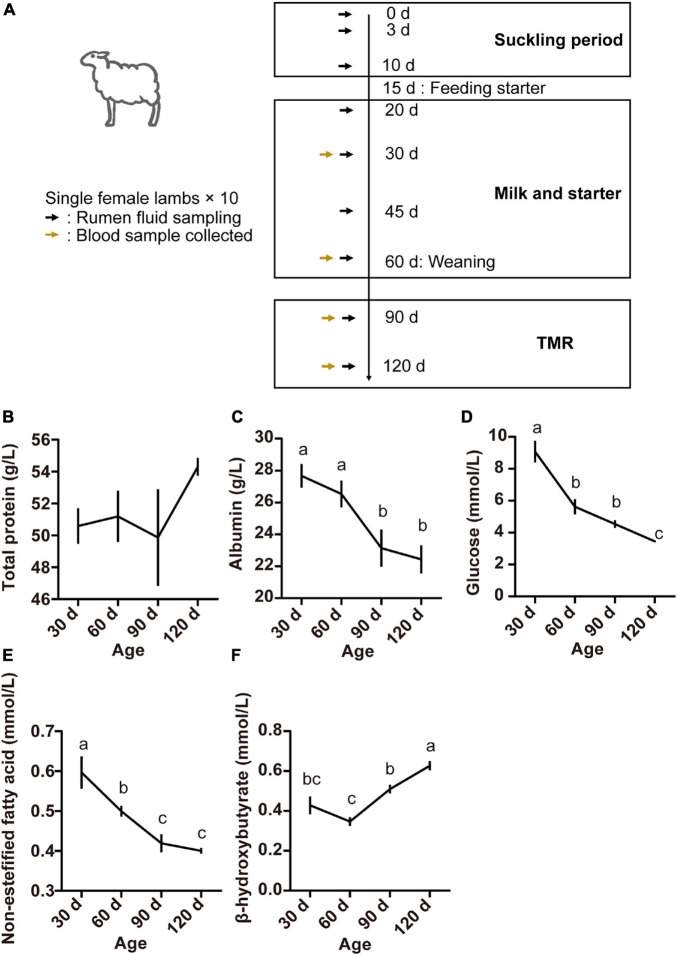
Experimental design and sampling schedule of lambs **(A)**, serum concentrations of total protein **(B)**, albumin **(C)**, glucose **(D)**, non-esterified fatty acids **(E)**, and beta-hydroxybutyric acid **(F)** at different lamb ages. Data are expressed as mean ± SEM. ^*a,b*^Letters denote significant differences between groups; groups that do not share the same letter are significantly different from each other (*P* < 0.05).

Blood samples (5 ml) were obtained from the lambs by jugular venipuncture on days 30, 60, 90, and 120 prior to the morning feeding (Figure 1A). They were allowed to coagulate, and the serum obtained by centrifugation at 3,000 × *g* for 15 min at 4°C was stored at −20°C prior to analysis.

The ruminal contents from each lamb were sampled at nine time points as follows: the first within 24 h after birth, then at the ages of 3, 10, 20, 30, 45, and 60 days before weaning, then at the ages of 90 and 120 days after weaning (Figure 1A). The rumen fluid samples were obtained 2 h after the morning feeding by use of an oral stomach tube, which was thoroughly cleaned using fresh warm water between sample collections (Ahmad et al., 2020). The rumen contents were collected into 20 mL cryopreservation tubes. All samples were immediately snap-frozen in liquid nitrogen and then stored at −80°C for further analysis.

### Sample Measurements

The analyses of the serum concentrations of total protein (TP), albumin (ALB), glucose (GLU), non-esterified fatty acids (NEFA), and beta-hydroxybutyric acid (BHBA) were performed by an automatic biochemical analyzer using standard commercial kits supplied from Nanjing Jian Cheng Bioengineering Institute (Nanjing, China).

The cryopreserved rumen fluid samples were thawed at 4°C and thoroughly mixed by vortexing. Later, 5 ml of the rumen fluid was taken and centrifuged at 3,000 × *g* for 10 min at 4°C. Then 1 ml of the supernatant was transferred to a 1.5-ml centrifuge tube containing 0.2 ml of metaphosphoric acid solution (25% w/v) with the internal standard 2-ethylbutyric acid. The mixture was placed in an ice-water bath for 30 min and centrifuged at 10,000 × *g* at 4°C. The resulting supernatant was transferred to a 1.5-ml centrifuge tube and stored at 4°C until analysis of the concentration of VFA.

The concentration of VFA was determined by gas chromatography (Varian 450, Agilent Technologies China, Co., Ltd., China) using the conditions and subsequent test procedures described earlier (Ahmad et al., 2020). Ammonia N concentration was determined spectrophotometrically using a colorimetric method (Weatherburn, 1967). The concentration of microbial crude protein (MCP) was determined by the Coomassie brilliant blue method (Bradford, 1976).

### DNA Extraction and Sequencing

The DNA of the rumen bacteria was extracted using the DNA Isolation Kit (MoBio Laboratories, Carlsbad, CA) following the manufacturer’s protocol. The quality of the obtained DNA was checked using 1% agarose gel electrophoresis and spectrophotometer (optical density at 260/280 nm ratio). We used universal primers to amplify the V3-V4 hypervariable regions of bacterial 16S rRNA gene. The primers were (338F: 5′- ACTCCTACGGGAGGCAGCAG −3′, 806R: 5′- GGACTACHVGGGTWTCTAAT −3′) (Munyaka et al., 2015). For each sample, a 10-digit barcode sequence was added to the 5′ end of the forward and reverse primers (provided by Allwegene Company, Beijing). The PCR was carried out on a Mastercycler Gradient (Eppendorf, Germany) using 25 μl reaction volumes, containing 12.5 μl KAPA 2G Robust Hot Start Ready Mix, 1 μl Forward Primer (5 μM), 1 μl Reverse Primer (5 μM), 5 μl DNA (total template quantity was 30 ng), and 5.5 μl H_2_O. Cycling parameters were 95°C for 5 min, followed by 32 cycles of 95°C for 45 s, 55°C for 50 s, and 72°C for 45 s with a final extension at 72°C for 10 min. Three PCR products per sample were pooled to mitigate reaction-level PCR biases. The PCR products were extracted from 2% agarose gels and purified using a QIAquick Gel Extraction Kit (QIAGEN, Germany) following the manufacturer’s instructions. Then they were quantified using QuantiFluor^TM^-ST (Promega, Madison, WI, United States). After that, the amplicons were sequenced at the Allwegene Company, Beijing. Deep sequencing of DNA extracts was performed on an Illumina Miseq PE300 sequencing platform (Caporaso et al., 2012) at the Allwegene Company (Beijing, China). After the run, image analysis, base calling, and error estimation were performed using Illumina Analysis Pipeline Version 2.6.

### Bioinformatics Analysis

The raw data were first screened, and the sequences that were shorter than 300 bp or had a low quality score (≤20), contained ambiguous bases, or did not exactly match primer sequences and barcode tags were removed from consideration. Only clean sequences with an overlap longer than 10 bp were assembled using FLASH-1.2.11 (Magoc and Salzberg, 2011). Reads that could not be assembled were discarded. Chimera sequences were detected using usearch6.1 (Edgar, 2010). Qualified reads were separated using sample-specific barcode sequences and trimmed with Illumina Analysis Pipeline Version 2.6. The resulting dataset was analyzed using QIIME1 pipeline (version 1.5.0). The sequences were clustered into operational taxonomic units (OTUs) at a similarity level of 97% (Edgar, 2013) to calculate the richness and diversity indices. The Ribosomal Database Project (RDP) Classifier tool was used to classify all the sequences into different taxonomic groups (Cole et al., 2009). The microbiota Bray- Curtis similarity was calculated based on OTUs. Chao 1 index and Shannon index, which reflect alpha diversity, were calculated by QIIME. Non-metric multidimensional scaling (NMDS) plots of the Bray-Curtis metric calculated with square root transformed data in R (vegan package). Heat maps were generated using the package “pheatmap” of R (v4.0.0) software. The functional profiles of bacterial communities were predicted by the method of Tax4Fun2 (Wemheuer et al., 2020). The 16S rRNA function prediction was categorized into the Kyoto Encyclopedia of Genes and Genomes (KEGG).

### RandomForest Analysis

We regressed (Subramanian et al., 2014) the relative abundances of bacterial taxa at the genus level along with the KEGG pathways against the ages of lambs using default parameters in the “RandomForest” package in R (Breiman, 2001) (ntree = 1,000, mtry = p/3, where p is the number of genera or the number of pathways) (Liaw and Wiener, 2002) in order to obtain the best discriminant performance of taxa and pathways across lambs’ ages. Lists of taxa and pathways ranked by RandomForest were performed in order of feature importance. Ten-fold cross-validation [the rfcv() function in the R package “RandomForest,” 10 repeats] was used to identify the number of marker taxa and pathways. After the minimum cross-validation error was obtained, we chose the number that stabilized against the cross-validation error curve as marker taxa and pathways correlating with the ages of lambs (Zhang et al., 2018).

### Data Availability Statement

The datasets generated from the current study are available in the Genome Sequence Archive repository^1^ under accession numbers PRJCA004193.

### Statistical Analysis

All statistical analyses were carried out using R (v4.0.0) software. A completely randomized design was used to analyze the results. An individual lamb was considered as an experimental unit for every analysis. The ruminal fermentation parameters and serum parameters were processed by ANOVA after checking independency, normality, and homogeneity:


Y⁢i=μ+X⁢i+e⁢i

where *Y*_*i*_ is the dependent parameter, *μ* is the overall mean, *X*_*i*_ is the age effect, and *e*_*i*_ is the residual error. Duncan’s multiple range test was used when significant effects were detected between ages. Spearman correlation between parameters was performed with CORR procedures. ANOSIM was performed to compare the similarity of bacterial communities in the rumen of different ages of lambs (Supplementary Data). Comparisons between groups were performed using a Wilcoxon test or Kruskal-Wallis test with R software. All data are presented as mean ± SEM. *P*-value < 0.05 was considered to indicate statistical significance.

## Results

### Blood Metabolites

Serum parameters for the different age groups are presented in Figure 1. The TP concentration was not affected by age (*P* > 0.05; Figure 1B). However, serum concentrations of ALB, GLU, and NEFA were higher at 30 days and declined thereafter (*P* < 0.05; Figures 1C–E). The concentration of BHBA increased from 60 to 120 days, finally reaching the highest at 120 days of age (*P* < 0.05; Figure 1F).

### Ruminal Fermentation Parameters

**FIGURE 2:**
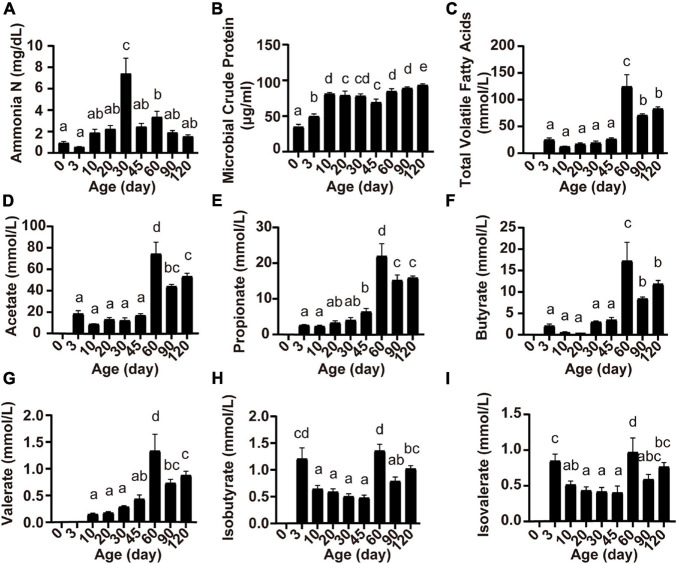
Serum concentrations of ammonia N **(A)**, microbial crude protein **(B)**, total volatile fatty acids **(C)**, acetate **(D)**, propionate **(E)**, butyrate **(F)**, valerate **(G)**, isobutyrate **(H)**, and isovalerate **(I)** at different lamb ages. Data are expressed as mean ± SEM. ^*a,b*^Letters denote significant differences between groups; groups that do not share the same letter are significantly different from each other (*P* < 0.05).

Ruminal fermentation parameters were affected by the age of the lambs (Figure 2). Rumen ammonia N concentration was highest at 30 days of age (*P* < 0.05; Figure 2A). The concentration of MCP increased from birth to 10 days of age and became relatively stable thereafter (*P* < 0.05; Figure 2B). Except for valerate which appeared at 10 days of age (Figure 2G), other volatile fatty acids (total VFA, acetate, propionate, butyrate, isobutyrate, and isovalerate) appeared at 3 days of age, and the concentration of all volatile fatty acids reached the highest at 60 days of age (*P* < 0.05; Figure 2).

### Changes in Rumen Microbiota and Taxonomic Composition

**FIGURE 3:**
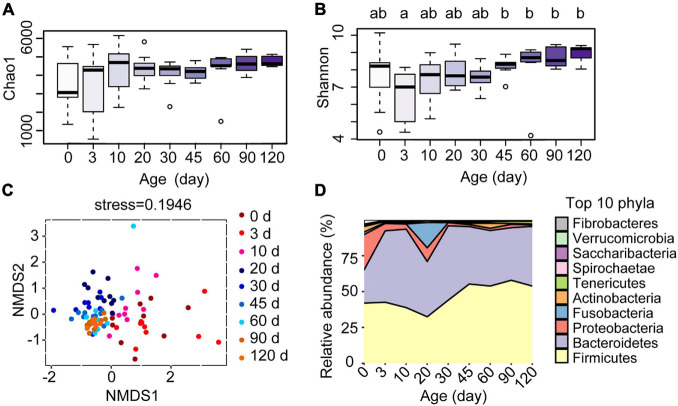
Effect of the age of lambs on rumen bacterial communities. **(A)** Species richness estimates (Chao1 index) and **(B)** diversity (Shannon index) for the nine sampling time points. **(C)** Scatterplot from non-metric multidimensional scaling (NMSD) of bacterial Bray-Curtis distance in each sample. **(D)** Relative abundance of bacterial phyla at different ages of the lambs. ^*a,b*^Letters denote significant differences between groups; groups that do not share the same letter are significantly different from each other (*P* < 0.05).

To investigate the development of the ruminal microbiota in these Hu sheep, we amplicon-sequenced the rumen fluid samples of the lambs from birth to 4 months of age (Figure 1A). The average number of high-quality rumen bacterial sequences generated per sample was 70,600. The Chao1 index, which reflects the species richness, increased after day 0 (Figure 3A), whereas the Shannon index, which indicates bacterial diversity, was observed lower at 3 days compared to the age groups of 45, 60, 90, and 120 days (Figure 3B, *P* < 0.05). The NMDS analysis, which compared the OTU community of each age group, revealed a high within-group similarity from 30 to 120 days of age (Figure 3C). The discrimination and high variance of samples collected at the 0, 3, and 10 age groups compared to later age groups confirmed the unique development of the bacterial community of each lamb before 10 days old. The samples collected at 20 days of age were also compositionally distinct to later samples but were similar to 30 days of age with less variance between ruminal samples. The significance of differences between groups was tested by analysis of similarity (ANOSIM, Supplementary Data). To further investigate the changes in specific rumen bacterial taxa during the early life of lambs, we compared the relative abundance of rumen bacteria at the phylum level (Figure 3D). The ruminal communities were dominated by Firmicutes (46.11%), Bacteroidetes (42.97%), and Proteobacteria (5.20%), accounting for 94.28% of all reads. From birth to 4 months of age, the relative abundances of Firmicutes and Bacteroidetes increased, whereas those of Proteobacteria and Fusobacteria decreased dramatically. The results showed that each age hosts its own particular bacterial community and the ruminal communities became more similar at the later time points.

### The Stability of Ruminal Microbiota Over Time

**FIGURE 4:**
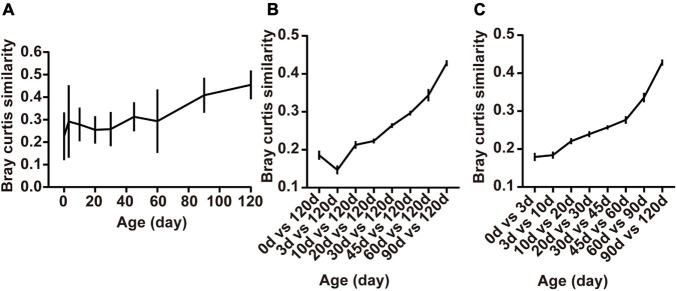
Changes in rumen bacterial diversity as the lambs age. **(A)** The Bray-Curtis similarity within groups, **(B)** between samples collected from birth to 90 days of age and those collected at 120 days of age, and **(C)** the similarity between adjacent time points. Data are expressed as mean ± SEM.

The within-group similarity comparison based on the Bray-Curtis distance of the bacterial communities among age groups declared a significant change in an age-dependent way (Figure 4A). The within-group similarity between different individuals increased significantly after birth with the continued increase of the alpha diversity (Chao1 and Shannon index), while no difference was observed in the inter-individual variation at 3 days compared to the age groups of 10, 20, 30, 45, and 60 days (Figure 4A, *P* < 0.05). The results of the within-group similarity demonstrated that the rumen bacterial ecosystem of lambs was exposed to a very high rate of inter-individual diversity and low stability before weaning.

Besides, ruminal samples of lambs apparently clustered together in the NMDS graph after 20 days, suggesting that the entire ruminal microbiota underwent relatively minor changes after 20 days of age and the community become relatively stable (Figure 3C). To highlight the process of progressive approximation to the community structure of the mature rumen microbiota in lambs, we determined the similarity between the rumen bacteria of samples collected from birth to 90 days of age and those collected at 120 days of age (Figure 4B). The results demonstrated that the composition of the lambs’ rumen bacterial ecosystem changed closer to the mature one, with a dramatic increase in the similarity after 20 days of age, reaching a maximum at 90 days vs. 120 days (*P* < 0.05, Figure 4B).

Furthermore, we determined the average Bray-Curtis similarity between adjacent age groups to describe the stability of an individual’s bacteria. The similarity illustrated that the bacterial composition of each individual’s rumen bacteria increased in an age-dependent manner. We found that rumen bacteria similarity between 0 and 3 days and 3 and 10 days was lower than the other groups. Meanwhile, the similarity between 90 and 120 days was the highest among the groups (*P* < 0.05, Figure 4C).

These results showed that large differences in bacterial community composition appear within the first few days after birth (days 0–3) and in the period shortly after providing starter feed (days 10–20). The bacterial communities changes fast during these periods, whereas a stable rumen bacterial community gradually becomes established after weaning (60 days of age).

### Age-Discriminatory Bacteria in Rumen Fluid of Lambs

**FIGURE 5:**
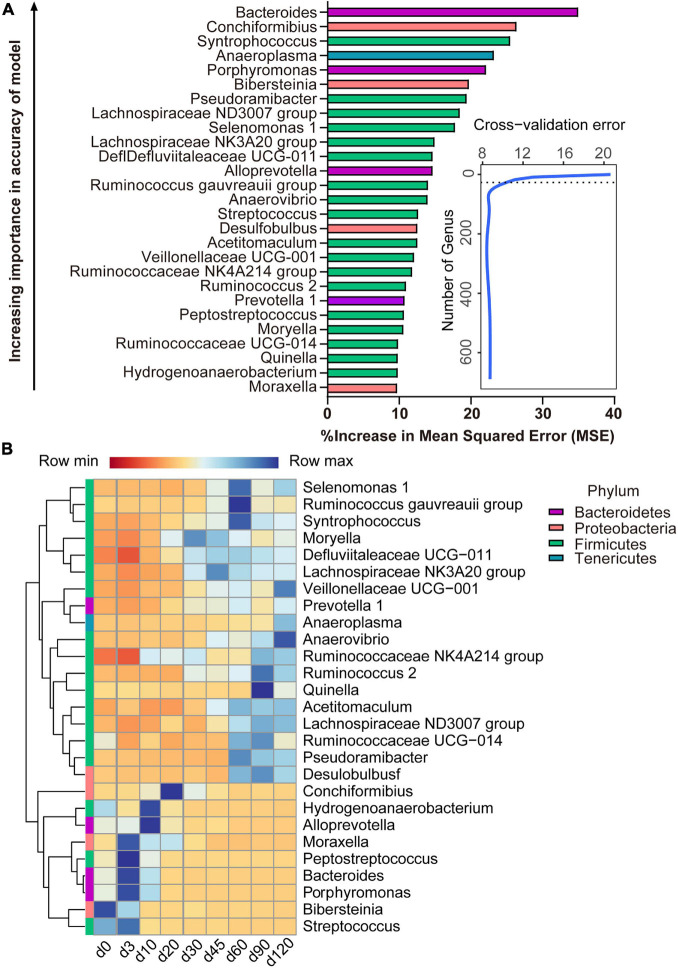
Bacterial taxonomic biomarkers of lamb age and the rumen microbiota gradually stabilize. **(A)** The top 27 biomarkers of bacterial genera were identified by applying RandomForest regression of their relative abundances in lambs against chronological age. Ranking in descending order of importance to the accuracy of the model was to determine biomarker taxa. The insert illustrates the result of the 10-fold cross-validation error. **(B)** Heatmap of changes over ages showing the relative abundances of the top 27 age-predictive biomarkers for bacterial genera.

With the application of the RandomForest algorithm to correlate rumen microbiota composition with ages, we found that the cross-validation error curve stabilized when 27 genera were used. Thus, these 27 genera are defined as biomarker taxa in the model and are shown in Figure 5A. All bacterial taxa were ranked in a descending order of importance. The age-discriminatory genera mostly belonged to Firmicutes (18/27), Proteobacteria (4/27), and Bacteroidetes (4/27) phyla. Of these age-discriminatory genera, 12 taxa (out of 27) increased abundance with the age of the lambs (*Veillonellaceae UCG-001*, *Prevotella 1*, *Anaeroplasma*, *Anaerovibrio*, *Ruminococcaceae NK4A214 group*, *Ruminococcus 2*, *Quinella*, *Acetitomaculum*, *Lachnospiraceae ND3007 group*, *Ruminococcaceae UCG-014*, *Pseudoramibacter*, and *Desulfobulbus*), 6 taxa obtained the highest relative abundance at 60 days of age (*Selenomonas 1*, *Ruminococcus gauvreauii group*, *Syntrophococcus*, *Moryella*, *Defluviitaleaceae UCG-011*, and *Lachnospiracea NK3A20 group*), the abundance of 6 taxa increased within the first 3 days after birth and decreased thereafter (*Moraxella*, *Peptostreptococcus*, *Bacteroides*, *Porphyromonas*, *Bibersteinia*, and *Streptococcus*), and 3 taxa increased in abundance up to 20 days of age and then decreased (*Conchiformibius*, *Hydrogenoanaerobacterium*, and *Alloprevotella*) (Figure 5B).

### Function Prediction of Rumen Microbiota

**FIGURE 6:**
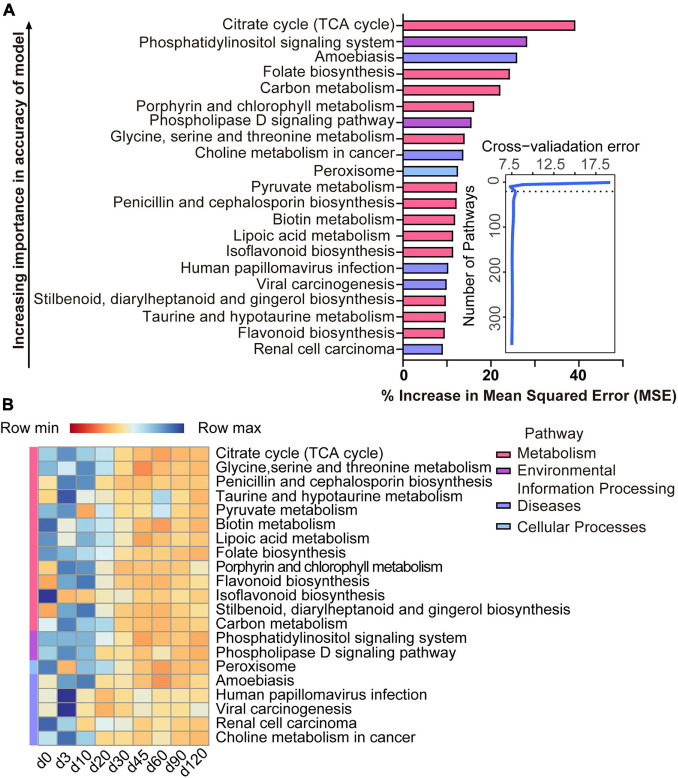
Bacterial pathway biomarkers of lamb age and the rumen microbiota gradually stabilize. **(A)** The top 21 biomarkers of bacterial pathways were identified by applying RandomForest regression of their relative abundances in lambs against chronological age. Ranking in descending order of importance to the accuracy of the model was to determine biomarker taxa. The insert illustrates the result of the 10-fold cross-validation error. **(B)** Heatmap of changes over ages showing the relative abundances of the top 21 age-predictive biomarkers for bacterial pathways.

The list of 21 pathways, in the order of time-discriminatory importance, which are defined as biomarker pathways in the model, is presented in Figure 6A (citrate cycle; glycine, serine, and threonine metabolism; penicillin and cephalosporin biosynthesis; taurine and hypotaurine metabolism; pyruvate metabolism; biotin metabolism; lipoic acid metabolism; folate biosynthesis; porphyrin and chlorophyll metabolism; flavonoid biosynthesis; isoflavonoid biosynthesis; stilbenoid, diarylheptanoid, and gingerol biosynthesis; carbon metabolism; phosphatidylinositol signaling system; phospholipase D signaling pathway; peroxisome; amoebiasis; human papillomavirus infection; viral carcinogenesis; renal cell carcinoma; choline metabolism in cancer). Most (13/21) of the important pathways are metabolic (including processes such as the citrate cycle and the metabolism of glycine, serine and threonine, taurine and hypotaurine, pyruvate, biotin, lipoic acid, porphyrin, and chlorophyll, and carbon), as well as biosynthetic processes (for penicillin, cephalosporin, folate, flavonoid, isoflavonoid, stilbenoid, diarylheptanoid, and gingerol). All of these pathways had a high relative abundance from birth to 10 days of age, which decreased thereafter (Figure 6B).

### Correlation Analyses of Age-Related Bacterial Genera and Functions With Rumen Fermentation and Serum Metabolite Levels

**FIGURE 7:**
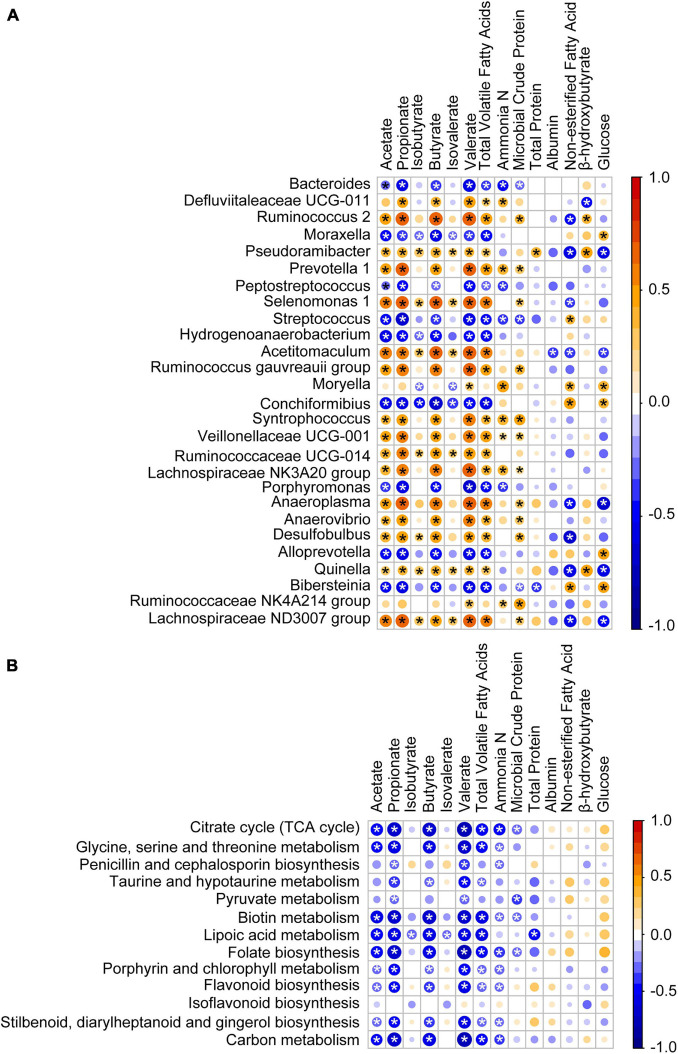
Association of the age-associated genera and pathways with lamb ruminal fermentation and blood serum parameters. **(A)** Spearman correlation of ruminal fermentation, serum parameters, and age-associated genera identified by RandomForest. **(B)** Spearman correlation of ruminal fermentation, serum parameters, and age-associated pathway identified by RandomForest. Bubble size represents the absolute value of the correlation coefficient (r), and bubble color represents negative (blue) or positive (red) correlations, *P < 0.05.

To investigate how the age-related genera and functions in rumen impact host metabolism, a Spearman correlation matrix was generated to explore these relationships. As shown in Figure 7A, the relative abundances of *Bacteroides*, *Moraxella*, *Peptostreptococcus*, *Streptococcus*, *Hydrogenoanaerobacterium*, *Conchiformibius*, *Porphyromonas*, *Alloprevotella*, and *Bibersteinia* had negative (*P* < 0.05) Spearman correlations with acetate, propionate, butyrate, valerate, and total VFA. In contrast relative abundances of *Ruminococcus 2*, *Pseudoramibacter*, *Prevotella 1*, *Selenomonas 1*, *Acetitomaculum*, *Ruminococcus gauvreauii group*, *Syntrophococcus*, *Veillonellaceae UCG-001*, *Ruminococcaceae UCG-014*, *Lachnospiraceae NK3A20 group*, *Anaeroplasma*, *Anaerovibrio*, *Desulfobulbus*, *Quinella*, and *Lachnospiraceae ND3007 group* were positively (*P* < 0.05) correlated with acetate, propionate, butyrate, valerate, and total VFA. Relative abundances of *Pseudoramibacter*, *Acetitomaculum*, *Anaeroplasma*, *Quinella*, and *Lachnospiraceae ND3007 group* were negatively (*P* < 0.05) correlated with NEFA and GLU and those of *Moryella*, *Conchiformibius*, and *Bibersteinia* were positively correlated with NEFA and GLU (*P* < 0.05). The relative abundances of the citrate cycle, the metabolism of glycine, serine and threonine, biotin, lipoic acid, the biosynthesis of folate, porphyrin, and chlorophyll, and carbon as well as of flavonoid, stilbenoid, diarylheptanoid, and gingerol were negatively correlated with acetate, propionate, butyrate, valerate, and total VFA (*P* < 0.05, Figure 7B).

## Discussion

In order to improve the efficiency of metabolic pathways, efforts have been made to investigate compounds that may modify key populations of microorganisms (Ahmad et al., 2020; Chen et al., 2020). However, because the rumen microbiota is generally well-established and hard to change by the time of such treatments, this approach has tended to be inconsistent or short-lived. Previous works have observed that the developing rumen in the newborn provides a unique opportunity to alter such a complex microbial ecosystem (Yá?ez-Ruiz et al., 2015; Abecia et al., 2018; Saro et al., 2018), but at this stage the most sensitive period of interventions on ruminal microbiota community and fermentation processes are not well known. The current study was designed to determine the effect of age on the rumen bacterial community and the ruminal fermentation of lambs. Both the composition and the fermentation of the microbial community were monitored from birth until lambs had access to adult diets. This study demonstrated that ruminal fermentation changed in an age-dependent manner that was correlated with some age-related alterations in rumen microbial taxa. It is important to know when modulating the composition and function of microbiota will be more effective. Our results indicated that it may be possible to manage these changes to improve rumen function and enhance the health and productivity of the host animals. In particular, these results suggest that the first 20 days from birth may be the most suitable period for the interventions.

The specific characteristic of ruminants is their ability to utilize the cellulose and hemicelluloses present in plant-based diets (Newbold and Ramos-Morales, 2020). In our study, as the lambs’ ruminal microbiota developed, we recorded changes in the relative abundances of Proteobacteria, Bacteroidetes, and Firmicutes that were consistent with previous studies (Jami et al., 2013; Zhu et al., 2018). The relative abundances of Firmicutes and Bacteroidetes increased, while that of Proteobacteria decreased dramatically. From 10 days on, there were no further dramatic changes of bacterial phyla indicating that, despite the fact that no solid plant material was ingested by the lambs, the bacteria responsible for its digestion were already present. Although the major types of rumen bacteria were present within 20 days, our findings on similarity suggested that the bacterial community was still unstable at this stage and thus was not fully matured. This supports the proposal that the period from birth to 20 days of age provides a unique opportunity to manipulate the composition of ruminal microbiota in lambs.

After 20 days of age, there were no clear age-related changes at the bacterial phyla level; however, there was variation in the relative abundance of some genera, which corresponds with changes of the substrate in the rumen (Yá?ez-Ruiz et al., 2015). Genera that were abundant from birth to 20 days of age in this and other studies (Dill-McFarland et al., 2017; Li et al., 2018; Yousif et al., 2018) included *Bacteroides*, *Moraxella*, *Peptostreptococcus*, *Streptococcus*, *Hydrogenoanaerobacterium*, *Conchiformibius*, *Porphyromonas*, *Alloprevotella*, and *Bibersteinia*, and these had a negative correlation with the main VFAs. These early rumen colonizers may have been spread from the birth canal or other environmental sources and are likely to be important for the newborn. For instance, the abundance of *Bacteroides* in newborn lambs enables them to utilize some components of milk (Mach et al., 2015). For the ruminal fermentation, the total volatile fatty acids appeared at 3 days of age, indicating that at the beginning of birth, though the microbiota already persists in the rumen, it was almost impossible to produce the SCFAs. Our results proved that the lambs can be considered a non-ruminant from a functional point of view in the early period (Jiao et al., 2016). Later, the microbiota present in the rumen will help the host to ingest feed. One of the important events in the development of microbiota is starter feeding, which can effectively facilitate the growth and development of the rumen. In our study, the similarity between the samples collected from birth to 90 days of age and those collected at 120 days of age increased after starter feeding (15 days of age). And after feeding solid food, the relative abundance of *Bacteroides*, which was important to digest milk (Mach et al., 2015), decreased. Our findings demonstrated that, in some way, the starter feeding affected the composition of ruminal bacteria which stabilized the bacterial ecosystem, consistent with a previous study in lambs (Lin et al., 2019). The presence of dietary fiber leads to a high abundance of *Prevotella* species (Kolodziejczyk et al., 2019). The butyrate-producing bacteria, Lachnospiraceae (*Lachnospiraceae NK3A20 group*) and Ruminococcaceae (*Ruminococcus gauvreauii group*, *Ruminococcaceae UCG-014*, and *Ruminococcus 2*) are critical for rumen fermentation (Ma et al., 2020). In our study, the age-related genera, *Prevotella 1*, *Lachnospiraceae NK3A20 group*, *Ruminococcus gauvreauii group*, *Ruminococcaceae UCG-014*, and *Ruminococcus 2*, all had a higher relative abundance after 30 days of age especially after weaning (60 days of age) along with a positive correlation with acetate, propionate, butyrate, valerate, and total VFA. Meanwhile, the concentrations of SCFAs significantly increased from 60 days of age. The changes of relative abundance in lambs showed that weaning will significantly increase the proportion of the bacteria which can produce the SCFAs, thus increasing the concentrations of SCFAs. The changes in the gene involved in the KEGG pathway also showed that the fermentation way in the rumen was changed after 20 days of age. The relatively oxidized rumen environment of newborn lambs enables rumen microbes to exploit the citrate cycle for energy production (Backhed et al., 2015) as shown by the enhanced expression of genes related to citrate cycle enzymes at this age. Our results illustrated that the microbiota which helps the neonate to utilize breast milk decreased, and then the microbiota which can ferment the solid food increased. Finally, a fully matured microbial ecosystem was established to digest plant material.

The feeding pattern will influence the bacterial communities in lambs. In our study, the lambs had free access to the starter from 15 days compared to a previous study in goats (free access to the starter from 25 days). The richness and diversity calculated by Chao and Shannon were higher in our study, and the relative abundance of Firmicutes and Bacteroidetes were different (Li et al., 2019). Compared to the Hu lambs that were weaned at 90 days, richness and diversity were higher in lambs 60, 90, and 120 days of age (Wang et al., 2016) in our study. The comparison of different feeding patterns indicated that both the time of feeding the starter and the time of weaning will affect the composition of the rumen bacterial community. Early intake of the starter and timely weaning will increase microbial diversity and make the microbial community mature earlier.

## Conclusion

This study of the ruminal microbiota of lambs shows that the bacterial diversity increases with the age of lambs and the age-related abundances of particular genera are correlated with concentrations of volatile fatty acids and microbial crude protein. The genera of *Prevotella 1*, *Lachnospiraceae NK3A20 group*, *Ruminococcus gauvreauii group*, *Ruminococcaceae UCG-014*, and *Ruminococcus 2* are important in maturation of both the rumen bacterial community and rumen fermentation. Although the composition and function of the ruminal microbiota are not well-established by 20 days of age of lambs, the major types of rumen bacteria are already present at this stage. This period provides a unique opportunity for the potential manipulation of the ruminal microbial ecosystems. The findings of the present study may be instructive for improving the ruminal fermentation processes and reprogramming the efficiency of rumen microbiota.

## Data Availability Statement

The datasets presented in this study can be found in online repositories. The names of the repository/repositories and accession number(s) can be found below: http://gsa.big.ac.cn, PRJCA004193.

## Ethics Statement

This study was conducted under the guidance of the Animal Care and Use Committee of Hebei Agricultural University (approval number: YJ201825).

## Author Contributions

XY: software, formal prioritized as targets in strength-based analysis, and writing—original draft. SJ: methodology, data curation, and term. CD: writing—review and editing. SJ and PT: investigation. HY: validation and visualization. YZ: conceptualization, project administration, and funding acquisition. YL: supervision and resources. All authors contributed to the article and approved the submitted version.

## Conflict of Interest

The authors declare that the research was conducted in the absence of any commercial or financial relationships that could be construed as a potential conflict of interest.

## Publisher’s Note

All claims expressed in this article are solely those of the authors and do not necessarily represent those of their affiliated organizations, or those of the publisher, the editors and the reviewers. Any product that may be evaluated in this article, or claim that may be made by its manufacturer, is not guaranteed or endorsed by the publisher.

## Footnotes

1
http://gsa.big.ac.cn
