# Supplementary material for: Age-Related Changes in the Ruminal Microbiota and Their Relationship With Rumen Fermentation in Lambs
Source: Front Microbiol. 2021 Sep 20;12:679135. doi: 10.3389/fmicb.2021.679135 (PMC8488279; doi:10.3389/fmicb.2021.679135)
Supplement: Supplementary file 1 [file Table_1.DOCX]

Age-related changes in the ruminal microbiota and their relationship with rumen fermentation in lambs

Xuejiao Yin^1†^, Shoukun Ji^1†^, Chunhui Duan^1^, Peizhi Tian^1^, Sisi Ju^1^, Hui Yan^1^, Yingjie Zhang^1*^, Yueqin Liu^1*^

^1^College of Animal Science and Technology, Hebei Agricultural University, Baoding 071000, P. R. China

**†** These authors have contributed equally to this work.

***** **Correspondence:**

Yingjie Zhang

zhangyingjie66@126.com

Yueqin Liu

liuyueqin66@126.com

Running title: Maturity of lambs’ ruminal microbiota.

**Supplementary Table S1**

Nutrient composition of experimental diets (dry matter basis)

| Items^1^, % | Starter | TMR |
| --- | --- | --- |
| Ingredients |  |  |
| Cornstalk |  | 40.00 |
| Corn | 53.20 | 35.50 |
| Soybean meal | 9.10 | 15.00 |
| Wheat bran | 2.00 | 7.20 |
| Soybean skin | 6.20 |  |
| Beet pulp | 4.70 |  |
| Extruded Soybean | 8.50 |  |
| Fermented soybean meal | 9.60 |  |
| [Soybean oil](http://dict.cnki.net/dict_result.aspx?scw=%e5%a4%a7%e8%b1%86%e6%b2%b9&tjType=sentence&style=&t=soybean+oil) | 2.20 |  |
| Whey powder | 2.00 |  |
| Limestone | 1.20 | 0.30 |
| CaHPO_4_ |  | 0.20 |
| NaCl | 0.30 | 0.80 |
| Premix^2^ | 1.00 | 1.00 |
| Total | 100.00 | 100.00 |
| Chemical compositions |  |  |
| Dry matter | 95.83 | 77.20 |
| Gross energy, MJ/kg DM | 17.56 | 15.07 |
| Crude protein | 21.20 | 14.61 |
| Neutral detergent fiber | 16.44 | 37.60 |
| Acid detergent fiber | 7.04 | 21.81 |
| Calcium | 0.74 | 0.70 |
| Phosphorus | 0.48 | 0.43 |

^1^Chemical composition measured by the AOAC method. TMR: Total Mixed Rations.

^2^Premix containing: 17456 IU/kg of vitamin A, 50 mg/kg of vitamin E, 3740 IU/kg of vitamin D, 98.70 mg/kg of Fe, 72.90 mg/kg of Zn, 57.4 0 mg/kg of Mn, 15.94 mg/kg of Cu, 0.33 mg/kg of Se, 1.30 mg/kg of I and 0.39 mg/kg of Co.
